# Advances in Multifunctional Nanoagents and SERS-Based Multimodal Sensing for Biotoxin in Foods

**DOI:** 10.3390/foods14081393

**Published:** 2025-04-17

**Authors:** Huan Jiang, Sihang Zhang, Bei Li, Long Wu

**Affiliations:** 1 Key Laboratory of Tropical Fruits and Vegetables Quality and Safety for State Market Regulation, School of Food Science and Engineering, Hainan University, Haikou 570228, China; jianghuan1210@163.com (H.J.); sih.zhang@foxmail.com (S.Z.); 2 Institute of Food Testing, Hainan Academy of Inspection and Testing, Haikou 570314, China

**Keywords:** food safety, mycotoxin, surface enhanced Raman spectroscopy, multifunctional nanoagent, multimodal biosensors

## Abstract

Biotoxins, toxic substances produced by living organisms, are widely present in food and pose a major threat to human health. Traditional detection methods, such as gas chromatography-mass spectrometry (GC-MS) and enzyme-linked immunosorbent assay (ELISA), often suffer from limitations including complex sample preparation, high costs, and lengthy analysis times. In response, surface-enhanced Raman spectroscopy (SERS) has emerged as a highly sensitive and specific analytical tool for the detection of biotoxins. This review highlights the recent progress in multimodal detection technologies based on SERS, focusing on the design and classification of multimodal materials to optimize the construction of SERS substrates. The integration of SERS with other detection modalities, such as fluorescence, colorimetry, and electrochemistry, is discussed to enhance the accuracy and diversity of biotoxin detection. Finally, the review critically assesses the current challenges and future prospects of SERS multimodal detection technology, particularly in real-time food safety monitoring and on-site diagnostics, offering critical insights to guide future research directions.

## 1. Introduction

Biological toxins in food, such as mycotoxins, bacterial toxins, and marine biotoxins [1], have become a serious challenge to global food safety because of their high toxicity, invisibility, and ubiquity. These toxins enter the human food chain through contamination of crops, aquatic products, and processed foods, leading to acute poisoning and chronic diseases such as hepatocellular carcinoma and immunosuppression [2], which are a major threat to public health. For example, aflatoxins are commonly found in moldy cereals and nuts [3]. Consumption of contaminated food may lead to vomiting, abdominal pain, and, in severe cases, liver cancer and even death. Botulinum toxin (BoNT) is one of the most toxic naturally occurring substances known [4], causing fatal respiratory failure even at trace concentrations. Marine biotoxins are naturally occurring, highly toxic biomolecules primarily found in marine organisms. These nonprotein toxic compounds are characterized by low molecular weight. Marine biotoxins such as tetrodotoxin (TTX), saxitoxin (STX), and okadaic acid (OA) are classified by their distinct chemical structures. Notably, these toxins may undergo structural modifications during food chain accumulation, potentially amplifying their toxicity [5]. When acting on the nervous or digestive systems, their potent toxicity and bioactivity can induce food poisoning symptoms ranging from diarrhea and paralysis to fatal outcomes [6]. The World Health Organization’s (WHO) 2021 Global Assessment of Carcinogens indicates that aflatoxin exposure is associated with nearly 150,000 annual hepatocellular carcinoma cases globally [7], of which approximately 30,000 cases specifically correlate with dietary exposure to contaminated sources. Furthermore, European Food Safety Authority (EFSA) 2023 statistics revealed 287 reported shellfish toxin contamination incidents across the EU in 2022, 62% of which were caused by saxitoxin, triggering emergency recalls of 43,000 metric tons of seafood products. These data emphasize the urgent need to strengthen food safety monitoring across both agricultural and aquatic supply chains. To address these risks, the development of rapid and accurate detection methods is imperative for enhancing regulatory frameworks and mitigating biotoxin-related health threats.

Chromatographic and immunoassay technologies provide multidimensional solutions for biotoxin detection. Gas chromatography (GC) and gas chromatography-mass spectrometry (GC-MS) employ gas-solid partitioning principles to analyze volatile compounds, demonstrating high separation efficiency and sensitivity for small-molecule toxins [8]. However, they require complex sample pretreatment and face thermal stability limitations. High-performance liquid chromatography (HPLC) employs liquid-solid phase interactions to accurately quantify nonvolatile toxins yet suffers from prolonged analysis time and high operational costs [9]. In the immunoassay domain, enzyme-linked immunosorbent assay (ELISA) achieves high-throughput screening with ultrahigh sensitivity through antigen-antibody binding coupled with enzymatic chromogenic reactions, whereas colloidal gold immunochromatography (GICA) enables rapid on-site detection via gold nanoparticle-based lateral flow assays [10,11]. Nevertheless, both immunoassays are limited by antibody cross-reactivity risks and semiquantitative capabilities. The inherent compromises between operational complexity, temporal efficiency [12], and analytical accuracy in conventional methodologies highlight the critical demand for innovative point-of-care testing (POCT) platforms.

Biosensors exhibit distinct advantages of high sensitivity, portability, low cost, and high efficiency [13]. Among them, surface-enhanced Raman spectroscopy (SERS) [14,15] colorimetric [16,17], fluorescence, and electrochemical biosensors have been extensively investigated for biotoxin detection. However, most nanomaterial-based detection techniques rely on a single signal readout, rendering these single-mode approaches susceptible to reliability issues in complex food matrices where interfering components may compromise signal interpretation and quantification accuracy [18]. For example, colorimetric signals are ineffective in dark food matrices [19], fluorescence signals struggle to overcome autofluorescence, and electrochemical stability and repeatability may be affected by temperature and humidity [20,21], leading to inconsistent results in practical applications.

**Figure 1:**
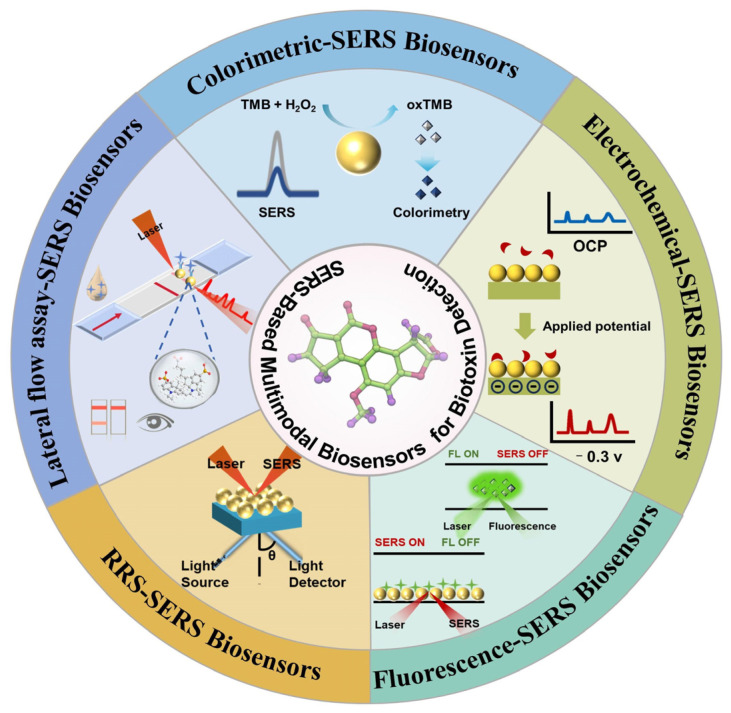
Schematic presentation of SERS-based multimodal biosensors for detection of biotoxins strategy design.

To address these challenges, novel nanomaterials have been strategically engineered to specifically detect target substances in food matrices while transducing detection events into multiplexed signal outputs [22], including fluorescence, colorimetry, temperature, and electrochemical responses. These advanced materials harness their intrinsic optical and physicochemical properties to integrate multimodal signal transduction pathways, thereby enabling self-verification mechanisms and self-repair functionalities [23,24], and are linked to portable devices such as smartphones and AI [25,26], along with a deep learning model that automatically extracts linear and nonlinear features from raw data. Emerging evidence indicates that deep learning algorithms significantly enhance spectroscopic applications in food quality assessment, improving both accuracy and robustness in toxin detection [27]. Multimodal detection strategies have emerged as transformative approaches [28,29,30], with their synergistic integration substantially enhancing detection precision for foodborne hazards without compromising rapid analysis capabilities. In this paper, we focus on multifunctional nanomaterials commonly used for biotoxin detection in food and review the research progress of multimodal SERS biosensors, including colorimetric-SERS, fluorescent-SERS, EC-SERS, and colorimetric-fluorescent-SERS, in the field of biotoxin research over the past 5 years. We systematically evaluate the advantages and limitations of SERS-based multimodal sensing platforms for biotoxin detection (Figure 1), while highlighting their transformative potential in biotoxin monitoring and food safety assurance.

## 2. SERS

### 2.1. Raman Spectroscopy

Raman spectroscopy, a technique based on inelastic light scattering from molecular vibrations, delivers unique chemical fingerprint signatures [31]. This method exhibits intrinsic advantages, including nondestructive analysis, label-free operation, and compatibility with aqueous samples, rendering it particularly promising for multicomponent analysis in complex matrices such as food systems. However, its applications are constrained by two critical limitations: the inherently weak Raman signal intensity, particularly for trace-level analytes such as ppm/ppb biotoxins in food, and interference from fluorescent backgrounds in complex matrices [32], which can overwhelm the target signal. These limitations critically hinder its real-world applicability in food safety surveillance.

### 2.2. SERS Substrate

SERS amplifies Raman signals by 10^6^–10^14^ times compared to conventional Raman spectroscopy, leveraging the localized surface plasmon resonance (LSPR) effect of precious metal nanomaterials (Au, Ag), thereby enabling ultrasensitive detection of trace toxins. Despite the significant advantages of SERS technology, its application in the detection of biotoxins in food is still subject to multiple limitations [33], including the difficulty of quantitative analysis due to the inhomogeneity of the substrate signals, the background interference caused by the complex food components, the nonspecific binding of nontarget molecules, and the susceptibility of noble metal nanoparticles to oxidation or aggregation that affects long-term stability.

Chemometrics has become indispensable in SERS spectral analysis. Through multidimensional data modeling, discriminative feature extraction, and advanced pattern recognition algorithms, chemometric approaches address critical challenges including signal intensity fluctuations, matrix interference, and nonlinear spectral responses, significantly enhancing the accuracy and reliability of both qualitative and quantitative analyses [34]. This synergistic integration is particularly impactful in food toxin detection, where high sensitivity and specificity are paramount. The deep convergence of chemometrics and SERS technology not only establishes a robust theoretical framework for trace analyte detection in complex matrices but also serves as a critical enabler for translating multimodal sensing platforms from laboratory prototypes to practical field applications [35]. Emerging innovations in materials science and detection strategies offer promising solutions to these persistent challenges.

The core driver of SERS performance is substrate design [36]. According to material composition and functional characteristics, SERS substrates are classified into three primary categories [37]: noble metal-based nanostructures, including spherical nanoparticles, core-shell architectures, and ordered nanoarrays, which predominantly amplify signals through electromagnetic enhancement (EM) mechanisms; semiconductor materials such as TiO_2_ and MoS_2_ that utilize chemical enhancement (CM) effects but necessitate integration with metallic components to attain sufficient sensitivity; and functionalized composite systems comprising magnetic Fe_3_O_4_@Au cores combined with molecularly imprinted polymer coatings, which enhance detection efficiency by simultaneously enabling target enrichment and selective molecular recognition.

Critical geometric parameters, specifically nanoparticle diameter and interparticle spacing, critically govern the intensity and spatial distribution of plasmonic “hot spots”. Although substantial advances in substrate engineering have been achieved, significant challenges persist in attaining the stability, consistent reproducibility, and homogeneous signal amplification essential for practical deployment of reliable detection systems in real-world scenarios.

Multifunctional nanomaterials significantly enhance SERS performance through modular designs that integrate molecular recognition, signal amplification, and interference suppression within a unified nanoplatform [38]. Aptamer-AuNPs exemplify this strategy by combining target-specific toxin capture with signal enhancement via tip-localized plasmonic effects [39], whereas Fe_3_O_4_@Ag magnetic nanocomposites coupled with an external magnetic field enable rapid target separation while minimizing matrix interference [40]. Additionally, core-shell architectures enhance substrate stability through physical isolation mechanisms. These innovations collectively address critical limitations of conventional SERS methodologies and establish a robust technological foundation for multimodal sensing paradigms.

## 3. Nanomaterials for SERS Sensors

### 3.1. Metal Nanomaterials

Metallic nanomaterials, particularly noble metals such as Au and Ag, exhibit distinctive optical, electrical, and catalytic properties that render them highly suitable for applications in chemical sensing, catalytic processes, and nanoscale device engineering [41]. The LSPR effect of metallic nanoparticles is critically influenced by key parameters including particle diameter, spatial orientation, geometric morphology, interparticle distance, dielectric properties, and surface characteristics [42,43]. Table 1 summarized the synthesis methods, diameters and performance evaluation of various nanomaterials in order to preliminarily explore the difference in detection performance of SERS substrates with different diameters. The distribution of the local electromagnetic field is not homogeneous, and there are regions at the nanoscale that have a high electromagnetic intensity (nanotips, interparticle nanogaps, and nanogaps of particulate matter) that are known as hot spots [44,45,46]. Anisotropic nanostructures such as nanorods, nanocubes, and nanotriangles with sharp geometric features exhibit enhanced capacity to generate intense localized electromagnetic fields under laser excitation [47]. Nanoclusters demonstrate exceptional potential for detecting biotoxins, pathogenic bacteria, and heavy metal ions due to their unique combination of low-energy electronic states, intense molecular fluorescence, PL, and superior structural stability [48].

Yu et al. developed a dual-mode aptasensor integrating fluorescence-SERS detection [49], utilizing gold nanoclusters (Au NCs) combined with silver nanoparticle-decorated metallic-polydopamine frameworks (Ag NPs/MPDA). This platform achieved ultrasensitive detection of deoxynivalenol (DON) in wheat flour with a limit of detection (LOD) of 0.06 ng/mL. Song et al. designed a colorimetric nanosensor utilizing papain-AuNCs and aptamers for specific identification of the foodborne pathogen *Escherichia coli* O157:H7 [50], demonstrating a linear detection range of 10^1^–10^6^ CFU/mL. Furthermore, Li et al. constructed a ratiometric fluorescence nanosensor by combining glutathione-functionalized silver nanoclusters (GSH-AgNCs) with a zirconium-based metal-organic framework (Zr-MOF), enabling trace-level Hg^2^⁺ detection in porphyra with a detection limit of 1.8 µg/L and a robust antimatrix interference capability [51]. These studies collectively demonstrate that nanocluster-based biosensors exhibit immense potential for food contaminant monitoring due to their operational simplicity, sensitivity, and selectivity.

When nanoparticle diameters fall within the 1–120 nm range, their physical, chemical, and electrical properties undergo significant modifications.A The nanoparticle diameter determines the LSPR wavelength, which must overlap with both the excitation laser wavelength and the Raman spectroscopy range to achieve optimal enhancement [52]. Increasing nanoparticle diameter induces a red shift in the surface plasmon resonance (SPR) peak. For instance, Ag NPs with diameters of approximately 15 nm exhibit characteristic plasmon resonance peaks near 400 nm. Nanoparticles in the 20–40 nm range generate intensified electromagnetic “hot spots” at interparticle junctions due to enhanced surface curvature effects, enabling single-molecule detection capabilities [53]. In contrast, larger particles (60–100 nm), though exhibiting weaker hot spot intensities, demonstrate superior monodispersity that facilitates quantitative analytical applications, as evidenced by rapid ochratoxin A (OTA) detection in maize matrices [54].

### 3.2. Metal-Organic Framework (MOF)

In recent years, MOFs have attracted significant research interest due to their ultrahigh porosity, large surface area, structural flexibility, and tailorable functionalities. As porous coordination polymers, MOFs effectively enhance the stability of SERS substrates [55,56]. Ren et al. developed a dual-mode aptasensor integrating colorimetric-SERS detection for specific identification of Shiga toxin type II (Stx2), utilizing a nanocomposite composed of noble metal nanoparticles and Raman reporter-loaded metal-organic frameworks (Mn/Fe-MIL(53)@AuNSs-MBA). The Mn/Fe-MIL(53)@AuNSs nanocomposite demonstrated dual functionalities: catalytic activity toward H_2_O_2_-mediated oxidation of 3,3′,5,5′-tetramethylbenzidine (TMB), enabling visual colorimetric detection through distinct chromatic transitions; SERS signal amplification via localized surface plasmon resonance (LSPR) enhancement from the anchored gold nanostars (AuNSs), which significantly intensified the Raman signature of the mercaptobenzoic acid (MBA) reporter molecules [57]. The exceptional SERS performance originated from the structural advantages of Mn/Fe-MIL(53), whose large pore size and high specific surface area facilitated dense AuNSs loading. This high-density AuNSs assembly created an intensive electromagnetic field coupling at interparticle gaps, thereby amplifying SERS signals by over 10^6^-fold compared to isolated nanoparticles. Furthermore, Wang et al. also constructed an SERS/fluorescence dual-mode sensor for the detection of enterotoxins in food using MIL-101 [58]. These developments collectively establish MOFs as pivotal components in multimodal sensing platforms, leveraging their synergistic signal enhancement, surface engineering versatility, and functional adaptability for high-performance toxin detection.

### 3.3. Covalent-Organic Framework (COF)

COFs are porous crystalline materials constructed from carbon (C), hydrogen (H), oxygen (O), boron (B), and nitrogen (N) interconnected through reversible covalent bonds [59]. COFs exhibit advantages including tunable pore architectures, high surface areas, and exceptional chemical stability [60]. Liang et al. employed dicyandiamide (Dd) and benzaldehyde (Bd) to create DdBd, a catalytically active COF material, to detect hygromycin residues in food; an SERS/RRS dual-mode sensor employing DdBd was built [61]. The surface electrons of DdBd sped up the production of AuNPs by accelerating the redox electron transfer process between Au^3+^ and polyethylene glycol 600 (PEG600). Additionally, DdBd accumulated more AuNPs, which significantly improved the SERS signal. These findings highlight COFs as a versatile class of tunable nanomaterials with extraordinary potential for advanced toxin-sensing applications.

### 3.4. Semiconductors

**Figure 2:**
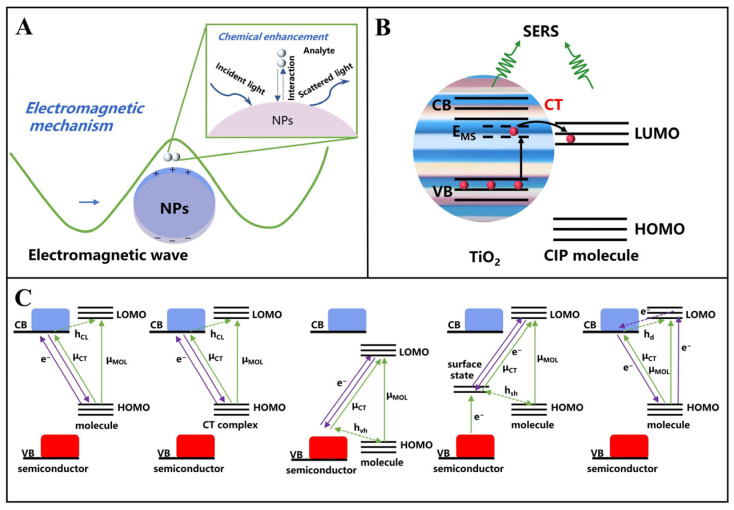
Schematic presentation of the principle of SERS enhancement: (**A**) Electromagnetic mechanism, (**B**) chemical enhancement, and (**C**) CT pathways in semiconductor molecule systems: molecule HOMO-to-CB, CT complex-to-CB, VB-to-molecule LOMO, surface state-to-molecule LOMO, and CB-to-molecule HOMO.

In recent years, semiconductor substrates have attracted growing research interest due to their exceptional chemical stability, superior biocompatibility, and controllable synthesis processes [62]. These substrates exhibit both EM and CM enhancement mechanisms in SERS applications, as schematically illustrated in Figure 2 [63]. Semiconductor-based SERS enhancement occurs through polarization and increasing the cross-section of Raman scattering by photoinduced charge transfer (PICT) between substrate and analyte, which modulates molecular polarization and increases the effective Raman scattering cross-section. Charge transfer (CT) in semiconductor-molecule systems can proceed via multiple pathways, including electron transitions from molecular HOMO to conduction band (CB), CT complex to CB, valence band (VB) to molecular LUMO, surface state to LUMO, and CB to HOMO.

Xu et al. propose a black TiO_2_ (B-TiO_2_)-based SERS bioprobe on a microfilter for the detection of circulating tumor cells (CTC) [64]. The SERS bioprobe was composed of crystal-amorphous core-shell B-TiO_2_ NPs, alizarin red (AR) as Raman reporter molecules, and a thin protective layer of NH_2_-PEG2000-COOH (PEG). Among these, the amorphous TiO_2_ structure improves PICT efficiency and generates a very high SERS enhancement factor (EF). In addition, Balaji et al. sputter-coated an Au layer on BiOBr for SERS-EC dual-mode detection of trichlorophenol (TCP) EF of the 10^7^ level was achieved [65]. These studies demonstrate that semiconductor substrates synergistically combine CM and EM mechanisms, enabling ultrasensitive detection in SERS applications through optimized enhancement strategies.

### 3.5. Two-Dimensional Materials

The boom of two-dimensional (2D) layered nanomaterials such as graphene, transition metal dichalcogenide (TMDC), hexagonal boron nitride (H-BN), and black phosphorus has opened up new avenues in SERS research based on CM [66,67]. These materials exhibit critical advantages, including structural homogeneity, chemical robustness, and efficient charge transfer capabilities [68]. For example, Raman-enhanced graphene has a typical layered structure, with the g-C_3_N_4_ nanosheets having relatively large surface areas filled with active electrons, which makes it easier to expose all functional groups and transfer charges [69]. Molybdenum disulfide (MoS_2_) exhibits a suitable CM enhancement due to its high electronic density states, and its composite structure was developed for the detection of miR-106a gastric cancer by SERS and electrochemical dual mode. Furthermore, Yao et al. loaded Ag nuclear Cu_2_O shells (Ag@Cu_2_O NPs) on MXene and fabricated a dual-mode SERS and EC sensor for detection of tetrodotoxin in foodstuffs [70]. MXene NSs had a good conductivity to accelerate the electrochemical oxidation process of Ag@Cu_2_O NPs and also had an inherent Raman signal, which could be used as a reference signal for calibration of SERS detection.

**Table 1:** Preparation and performance characterization of SERS substrates (Note: /, not reported).

Material	Synthesis	Material Diameter (nm)	Target	AEF	Linear Range	LOD	Ref.
cDNA-AuNCs AgNPs/MPDA-Apt-TAMRA	Covalent Conjugation Thiol-Metal Covalent Conjugation	3.06 ± 0.70 (AuNCs) 27.40 ± 2.48 (AgNPs)	DON	2.1 × 10^6^	0.1–100 ng/mL	0.06 ng/mL	[49]
aptamers@papain@AuNCs	Biotemplated Synthesis Thiol-Metal Covalent Conjugation	10.17 ± 1.21	*Escherichia coli* O157:H7	/	10–10^6^ cfu/mL	39 cfu/mL	[50]
AgNCs Zr-MOF	Chemical Reduction Synthesis Solvothermal Synthesis	2–5 100–200	Hg^2+^	/	10–500 ng/mL	1.8 ng/mL	[51]
Fe_3_O_4_@SiO_2_@Ag-Apt AuNPs-cDNA	Seed-Mediated Growth Thiol-Metal Covalent Conjugation	500 50	OTA	1.57 × 10^7^	0.01–5 ng/mL	0.0074 ng/mL	[54]
MBs-apt Mn/Fe-MIL (53)@AuNSs-MBA-cDNA	Biotin-Avidin System Electrostatic Self-Assembly	/	Stx2	/	0.05–1000 ng/mL	0.026 ng/mL	[57]
DdBd AuNPs	Solvothermal Polycondensation Nanocatalytic Analysis	70 30	OTC	3.67 × 10^6^	0.0012–0.028 ng/mL	3.6 × 10^−4^ ng/mL	[61]
B–TiO_2_-AR-PEG-FA	Solid-phase synthesis	25 (B–TiO_2_ NPs)	CTC	/	/	2 cells/mL	[64]
2D BiOI	Hydrothermal	120	TCP	10^7^	1.97–32,770 ng/mL	0.0197 ng/mL	[65]
Ag@Cu_2_O NPs MXene NSs	Chemical Reduction Chemical Etching	80.1	TTX	/	0.1–10^4^ ng/mL	0.0316 ng/mL	[70]

## 4. Applications of SERS-Based Multimodal Sensors

SERS has made a series of advances in the detection of biotoxins in food [71]. However, in addition to constructing multifunctional nanomaterials to address challenges in SERS quantitative analysis [72], multimodal detection strategies can be employed to enhance data reliability and accuracy. SERS multimodal sensors, which integrate SERS with electrical, electrochemical, acoustic, magnetic resonance spectroscopy (MRS), colorimetric, and fluorescence technologies [73], enable cross-validation through independent multiresponse signals, thereby improving detection accuracy compared to single-mode approaches. The availability of multiple data sources combined with multisource data fusion presents promising research prospects. The innovative modular design realizes the organic integration of “recognition-enhancement-anti-interference” functions [74], which drives the advancement of detection technologies toward intelligent and portable systems.

### 4.1. SERS-Colorimetric Sensors

**Figure 3:**
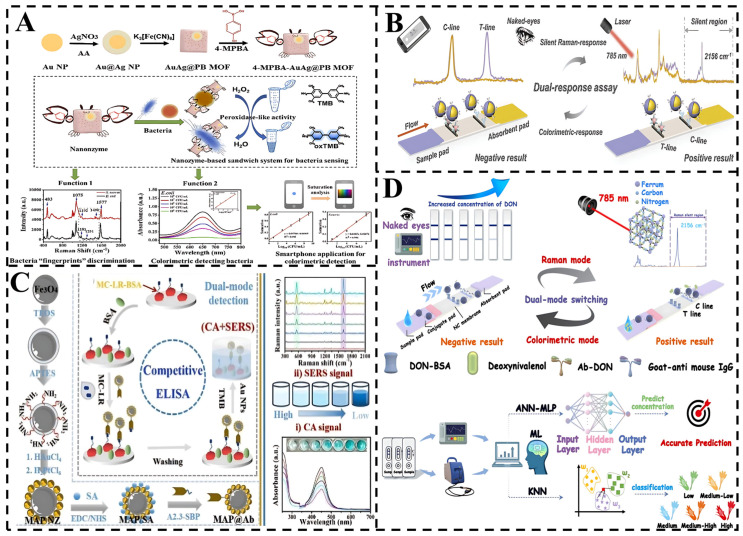
(**A**) Schematic illustration of a nanozyme-based sandwich system using bacteria/4-MPBA/. AuAg@PB MOF [77]. (**B**) Dual-response mediated ECCRD assay using APNPs-LFIA [78]. (**C**) Schematic illustration of an immunosensor for rapid detection of MC-LR based on MAP@Ab probes catalyzed TMB [79]. (**D**) The LFIA system and its ML-Optimized LFIA Colorimetric and SERS Analysis of DON Classification and Prediction in Grain [80].

Among the reported bimodal strategies, the SERS-colorimetric method is widely used for the detection of food toxins (Figure 3). This approach achieves a wide detection range through the integration of different reporting units [75], with SERS reporting providing molecular specificity information and colorimetric reporting measuring absorbance and transmittance to characterize light-sample interactions [76]. Integration of these reporting units into a hybrid system captures different sample characteristics, including chemical bonding as well as the concentration and optical properties of the analyte, thus increasing the overall bimodal accuracy, sensitivity, and specificity. Meanwhile, smartphone integration as a colorimetric reader with SERS substrates enables analyte quantification through color changes, which can be further extended to portable platforms by incorporating 3D-printed accessories for target detection.

These advances in bimodal detection strategies highlight the versatility and practicality of combining SERS with colorimetric techniques. For instance, Cai et al. synthesized a multifunctional nanoenzyme (AuAg@PB MOF) with colorimetric-SERS signaling, which acted as an SERS probe to enhance the Raman signal [77]. A 4-MPBA molecule acting as an SERS tag modified on the AuAg@PB MOF via Ag–S bonding, and 4-MPBA coupled to the bacteria by binding to bacterial sugars. This formed a sandwich structure for trapping bacteria, providing a new alternative method for rapid and accurate bacterial detection.

Furthermore, Liu et al. optimized this approach by developing a multifunctional core-shell nanotracer (APNPs). This system exhibited significantly enhanced sensitivity in the colorimetric-SERS response compared to traditional single-modal methods, such as AuNPs-lateral flow immunoassay (AuNPs-LFIA), as well as other control groups [78], offering an efficient tool for the monitoring of ECCRD in food safety. Moreover, Wu et al. engineered a bifunctional nanobody (A2.3-SBP) with dual-binding capability for microcystin-leucine-arginine (MC-LR) and streptavidin [79]. By leveraging this nanobody alongside Fe_3_O_4_@Au-Pt nanozymes, they developed a dual-mode colorimetric-SERS platform for MC-LR detection. The system exhibited linear responses between MC-LR concentrations and both chromatic transitions and SERS peak intensities, achieving ultralow detection limits of 0.26 ng/mL and 0.032 ng/mL, respectively. Notably, the rapid synthesis, cost-effectiveness, and exceptional stability of the Fe_3_O_4_@Au-Pt nanozymes position this dual-mode platform as a robust solution for monitoring MC-LR and structurally analogous cyanotoxins in aquatic environments.

Further advancing the field, Sun et al. developed a dual-mode colorimetric-SERS LFIA for detecting DON [80]. The rhodium nanocores provided strong plasmonic properties as the SERS substrate, while AgNPs created electromagnetic “hotspots” to enhance signal sensitivity. Finite element modeling was used to optimize the electromagnetic field intensity. This dual-mode LFIA achieved a detection limit of 4.21 pg/mL, 37-fold lower than that of colloidal gold-based LFIA (0.156 ng/mL). Machine learning algorithms, including ANN and KNN, enabled precise classification and quantification of contamination, achieving 98.8% classification accuracy and a mean square error (MSE) of 0.57 pg/mL. This work offers a new path for designing nanozyme-based SERS protocols for food analysis.

These results underscore the potential of the colorimetric-SERS platform for analyzing harmful substances in complex matrices and demonstrate the crucial role of machine learning-enhanced nanosensors in advancing detection technologies. The SERS-colorimetric dual-mode detection technology exhibits unique application value in food biotoxin detection by integrating the advantages of rapid primary screening and accurate verification. In the future, with deeper integration of portable equipment, smart materials, and AI technology, this strategy is expected to become a core tool for on-site food safety monitoring and to facilitate rapid technology transfer from the laboratory to the industrial chain.

### 4.2. SERS-Fluorescence Assay

**Figure 4:**
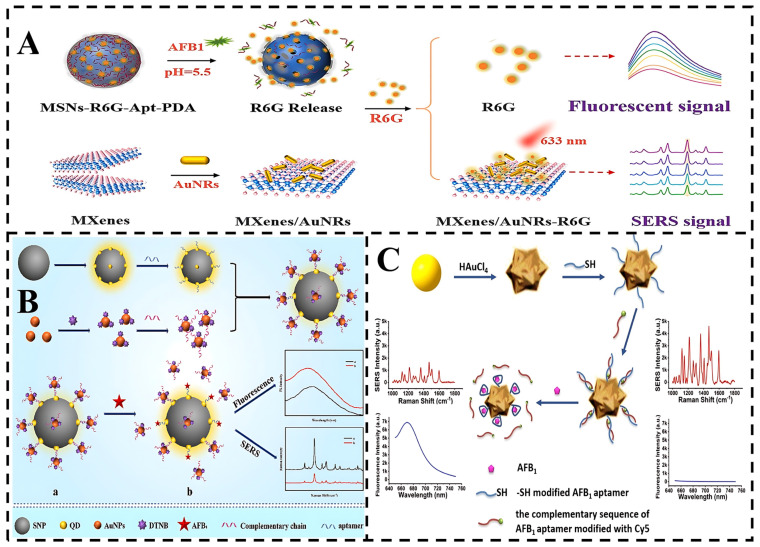
(**A**) Schematic illustration of the dual signal-on biosensor for SERS and fluorescence detection of AFB1 [83]. (**B**) Schematic diagram of the synthesis process for Raman and fluorescence dual-mode aptasensor and its application in AFB1 detection [84]. (**C**) Schematic illustration of the SERS/fluorescence dual-mode nanosensor based on AuNFs for AFB1 detection [86].

Fluorescence spectroscopy provides rapid optical visualization of samples and multiple detection of fluorophores using multiple fluorescence channels [81]. SERS mapping provides highly specific spectral information based on the chemical properties of the molecules present [82]. Therefore, the SERS-fluorescence technology combines molecular SERS specificity and rapid fluorescence visualization to increase the sensitivity and accuracy of the test (Figure 4). In a typical SERS fluorescence bimodal system, the substrate consists of fluorescent nanoparticles or dyes for fluorescence imaging and metal nanoparticles for enhancing the SERS signal.

As an example, Wu et al. used aminated mesoporous silica nanoparticles (MSNs) as nanocontainers loaded with rhodamine 6G (R6G) with dual fluorescence and SERS effects, where the degradation of polydopamine and the specific binding of aptamers to aflatoxin B1 (AFB1) triggered the release of R6G, producing fluorescence and SERS intensity changes after the addition of AFB1 under acidic conditions [83]. Similarly, Wei et al. constructed a dual-signal SERS-fluorescence aptasensor using the SPR and FRET properties of quantum dots and AuNPs [84]. When the SERS nanotags are clustered around the QD-SNPs, the detection system generates stronger SERS signals and significantly suppressed fluorescence signals, as the rational clustering of SERS nanotags creates additional “hot spots” and enhances resonance energy transfer. Owing to the strong affinity between the aptamer and the target molecule, the presence of AFB1 displaces the SERS nanotag in the satellite nanostructure, leading to a weakened SERS signal and a progressive increase in fluorescence intensity. Furthermore, the fluorescence-SERS assay results were validated against HPLC, achieving recoveries within the reliable range of 92.75–105.24%, which validates the potential of SERS-based multimodal biosensors for real-sample detection.

Nanoclusters have recently emerged as luminescent nanomaterials with practical applications because of their unique optical and electrochemical properties [85]. In another innovative design, Song et al. designed a gold nanoflowers (AuNFs) detection of AFB1 [86]. AuNFs exhibited both excellent SERS enhancement and strong fluorescence quenching effects, enabling dual-signal detection. Then, the quantitative detection was realized with two optical properties. The LOD was calculated to be 0.03 ng/mL. Compared to single-mode aptasensors, the dual-mode aptasensor offers enhanced reliability, flexibility, and anti-interference capability.

The results demonstrated that the SERS-fluorescence dual-mode detection technique exhibits high sensitivity, reliability, and anti-interference capabilities in food biotoxin detection by integrating the quantitative power of fluorescence with the molecular fingerprint specificity of SERS. Furthermore, simultaneous detection of multiple toxins can be enabled using fluorescence-SERS nanoparticles encoded with different colors, coupled with deep learning algorithms, expanding their commercial applicability.

### 4.3. EC-SERS Detection

**Figure 5:**
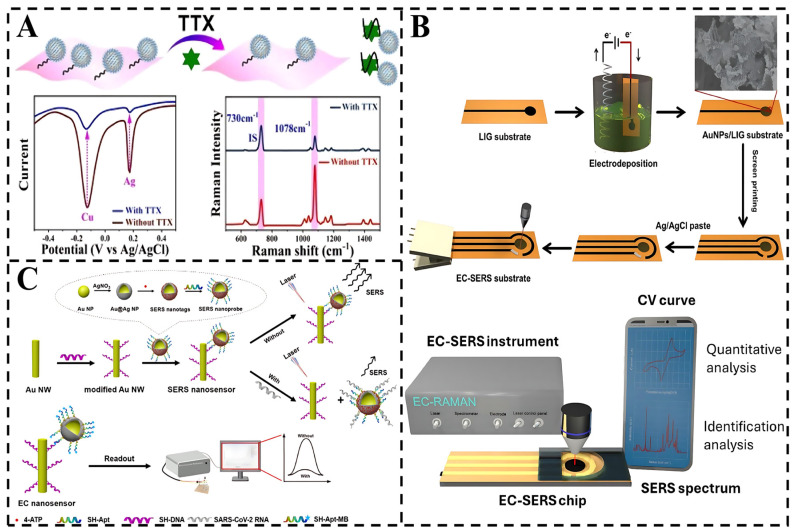
(**A**) Schematic diagram for detection of TTX based on Ag@Cu_2_O NPs label and MXene nanosheets [70]. (**B**) Preparation process for EC-SERS substrates and schematic of the chip for uremic toxin detection [88]. (**C**) Schematic illustration of the SERS/EC-based nanosensor [89].

EC-SERS integrates electrochemical modulation with an SERS-active substrate, where the working electrode, based on noble metal modification, serves as a bifunctional platform for simultaneous electrochemical control and Raman signal enhancement, enabling real-time, sensitive detection. EC-SERS acts on two main technological mechanisms [87]: First, the applied potential changes the charge state of the electrode surface, regulating the adsorption orientation or redox reaction of the target molecules and optimizing their distribution in the SERS active sites (“hot spots”). Secondly, the Raman signal is amplified by LSPR with an EF of 10^8^–10^12^. The EC-SERS sensor not only monitors in real time the molecular conformational changes during electrochemical reactions, such as the generation of toxin redox intermediates. It is also able to selectively excite the redox reactions of target molecules by means of potentiostatic excitation, excluding the influence of nonelectrically active interferents (Figure 5).

For instance, Yao et al. designed dual-mode nanotags with noninterfering sensing signals to improve the detection accuracy and sensitivity of TTX. Electroactive and SERS-active Ag@Cu_2_O NPs were fabricated. The dielectric Cu_2_O shell with a large dielectric constant inhibited the attenuation of EM waves of Ag NPs, which strengthened the EM fields for SERS enhancement and sped up electron transfer. The results show that the LODs are 31.6 pg/mL for the electrochemical signal and 38.3 pg/mL for the SERS signal.

Similarly, Juang et al. developed a rapid and sensitive method for detecting uremic toxins [88]. The Laser-Induced Graphene (LIG) substrate provides a stable platform for AuNPs, facilitating interaction with target molecules. Additionally, electrochemical deposition optimizes the sensor’s sensitivity by amplifying the local electromagnetic field around AuNPs and offering specific binding sites for uremic toxins. The sensor demonstrates exceptional performance, achieving low LOD for creatinine, uric acid, and urea, and offering concentration-dependent responses in cyclic voltammetry measurements. These findings on EC characteristics that enhance SERS signals open new avenues for developing multifunctional EC-SERS sensors for biotoxin monitoring.

Dai et al. developed a dual-mode sensing system combining SERS and EC using single gold nanowires (Au NWS). By modifying Au NWs with bimetallic nanoparticles [89], the SERS/EC signals are significantly enhanced. The presence of SARS-CoV-2 RNA interacts with aptamers containing signal molecules, leading to a notable attenuation of the SERS/EC signals. The nanosensor demonstrates remarkable specificity for SARS-CoV-2 RNA detection and high sensitivity, with LODs of 1.02 pM by SERS and 0.21 pM by EC methods. Critically, its successful application in complex matrices validates its potential as a reliable tool for toxin detection.

In conclusion, EC-SERS technology achieves a dual breakthrough in sensitivity and selectivity for food biotoxin detection through the synergistic integration of electrochemistry and SERS while offering unique advantages in real-time dynamic analysis. The development of a miniaturized EC-SERS chip, integrated with a three-electrode system and a wireless data transmission module, combined with a smartphone application for real-time display of spectral and electrochemical data, can achieve a high degree of dynamic adaptation to the detection of different food matrices.

### 4.4. SERS-RRS Technique

**Figure 6:**
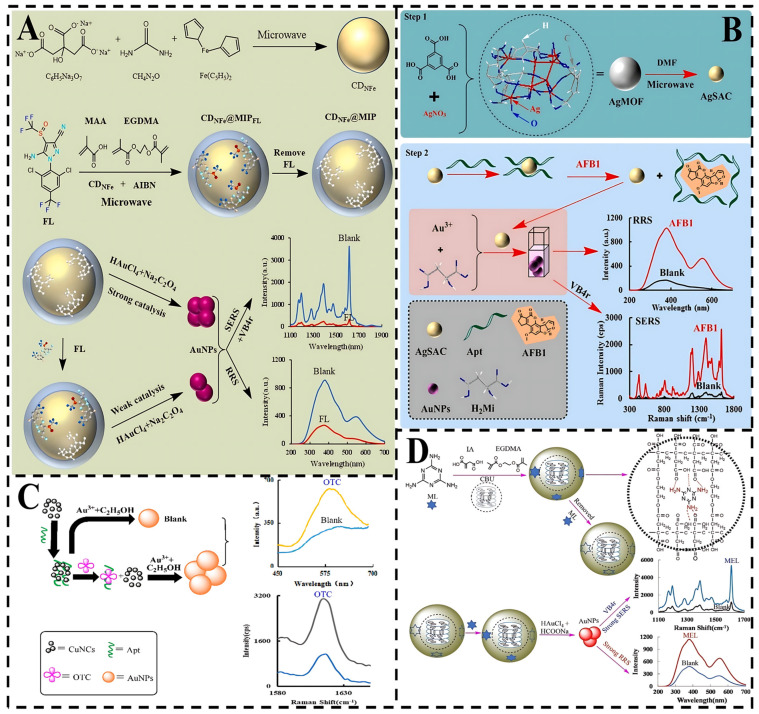
(**A**) On-site generation of nanogold for dual-mode FL detection based on CDNFe@MIP-catalyzed sodium oxalate reduction of chloroauric acid [93]. (**B**) Principle of aptamer-mediated SERS/RRS for AFB1 based on AgSAC catalysis [94]. (**C**) Schematic diagram of assay of OTC based on aptamer controlling CuNCs catalysis [95]. (**D**) Schematic of the detection ML of AuNPs formed from SF reduction of HAuCl_4_ catalyzed by CBU@MIP [96].

Resonance Rayleigh scattering (RRS) is an elastic light scattering phenomenon [90]. Owing to its ability to provide structural information about molecules, including medium composition, size, shape, charge distribution, and refractive index, RRS has been widely employed in the design and development of various analytical platforms [91]. In contrast, SERS is an inelastic light scattering technique [92]. The dual-spectral analysis method combining SERS and RRS can not only verify and complement each other but also provide two new sensitive detection methods (Figure 6).

For instance, Liu et al. study N- and Fe-doped carbon atoms (CDNFe) have been synthesized by microwave synthesis [93]. Utilizing CDNFe as a nanosubstrate, fipronil (FL) as a template, and α-methacrylic acid as a functional monomer, a dual-functional molecular imprinted polymethacrylic acid nanoprobe (CDNFe@MIP) was fabricated through microwave processing, which revealed its ability to selectively recognize FL and catalyzed the HAuCl_4_-Na_2_C_2_O_4_ nanogold-based indicator reaction. Notably, FL recognition reduced the catalytic effect of CDNFe@MIP. Leveraging the SERS and RRS properties of AuNPs generated from HAuCl_4_ reduction, a dual-mode sensing platform for FL detection (5–500 ng/L) was developed. The molecularly imprinted nanoprobes on the surface of these core-shell structure-doped carbon dots exhibit high catalytic amplification and specific recognition, suggesting their potential for the detection of a wide range of toxins in practical applications.

Similarly, Lv et al. synthesized a highly active silver single-atom catalyst (AgSAC) [94]. AgSAC demonstrated exceptional catalytic performance in catalyzing the reaction between chloroauric acid-malic acid (HAuCl_4_-H_2_Mi) to produce AuNPs with intense SERS, RRS, and surface plasmon resonance absorption (Abs) signals. The catalyst’s activity could be modulated by AFB1 aptamer adsorption, reducing the SERS/RRS/Abs signals. In the presence of AFB1, specific binding to the AFB1 aptamer released AgSAC, restoring its catalytic activity and signals. These high-performance optoelectronic active materials based on MOFs provide new insights for RRS-SERS detection of biotoxins.

Furthermore, Chen et al. established a novel method for detecting oxytetracycline (OTC) by coupling the catalytic amplification reaction of copper nanoclusters (CuNCs) with aptamer recognition. CuNCs have the catalytic activity for the formation of AuNPs resulting from a HAuCl_4_-ethanol (En) reaction [95]. The OTC aptamer nonspecifically adsorbs onto CuNCs, inhibiting their catalytic activity. Upon OTC addition, the aptamer-OTC complex forms, causing CuNCs to desorb and restore catalytic function. The generated AuNPs exhibited SERS signals at 1615 cm^−1^ in the presence of Vitoria blue 4R (VB4R) molecular probes and an RRS peak at 586 nm. This method combines simplicity, rapidity, stability, and sensitivity, offering a new approach for biotoxin detection.

Additionally, Yi et al. developed a novel two-mode scattering spectroscopy method for the rapid detection of ultratrace melamine (ML) in dairy products [96]. Specifically, cholesteryl butyrate (CBU) nanosurface imprinted polymers (CBU@MIP) were utilized, which not only recognized ML but also catalyzed the formation of Au NPs from HAuCl_4_ and sodium formate. These AuNPs displayed SERS and RRS effects. Upon ML addition, CBU@MIP specifically bound to ML, forming conjugates with enhanced catalytic activity. This led to a linear increase in SERS and RRS signals, with LODs of 0.0072 pmol/L and 0.093 pmol/L, respectively. The CBU@MIP design demonstrates significant potential for constructing SERS-RRS sensors for biotoxin detection.

Collectively, the above studies demonstrate that integrating SERS and RRS provides an efficient, sensitive, and versatile analytical platform for biotoxin detection. This approach can further be combined with multifunctional encoded nanoparticles and deep learning algorithms to enable simultaneous multitoxin detection and automated spectral analysis, particularly for complex sample matrices in future applications.

### 4.5. Other SERS-Based Techniques

**Figure 7:**
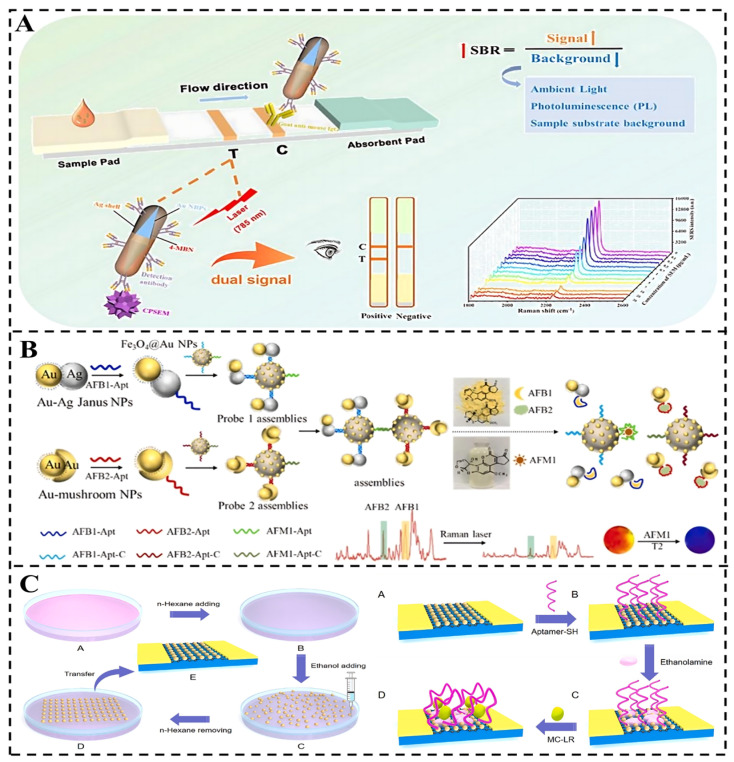
(**A**) Operational principle of semiquantitative visual and quantitative detection using SERS-LFIA [99]. (**B**) Schematic illustration of aflatoxin subtype analysis based on label-free SERS nanoprobes and magnetic nanoprobes [100]. (**C**) Immobilization process of MC-LR aptamer on AuNP/graphene/glass substrate for MC-LR detection [101].

In addition to the integration techniques mentioned above, current research includes the use of LFIA, SPR, field effect transistors (FET), MRS, thin-layer chromatography (TLC), etc. (Figure 7). in conjunction with SERS. In particular, LFIA strips are available for immediate application of the test [97], SPR offers high sensitivity, label-free and real-time monitoring capability [98], FET is a simple and effective tool for simulation, MRS detects magnetic changes that are correlated with biotoxins, and TLC is a simple and effective tool for initial screening. These integrated techniques enhance detection accuracy and reliability while also facilitating a more comprehensive understanding of biotoxin chemical structures, interactions, and potential effects.

For instance, Tian et al. developed an ultrasensitive LFIA that combines SERS/colorimetric dual-signaling modes for detecting nitrofurazone metabolites [99]. The assay employed Au@4-MBN@AgNRs nanosandwich-structured signal tags, leveraging high signal-to-background ratios and matrix interference resistance through geometric and chemical nanoengineering. Optimal conditions yielded LODs of 20 pg/mL (colorimetric) and 0.08 pg/mL (SERS). Notably, the colorimetric detection limit was enhanced by 100-fold compared to Au NPs-based LFIA. Likewise, Cao et al. designed a dual-mode sensor integrating SERS and MRS signals that has been devised for critical monitoring of aflatoxin subtypes AFB1, aflatoxin B2 (AFB2), and AFM1 [100]. This approach employs Au-Ag Janus NPs and Au-mushroom NPs as SERS nanotags for AFB1 and AFB2 detection, exhibiting intense, noninterfering SERS peaks. Fe_3_O_4_@Au NPs functionalized with AFM1 aptamers serve as MRS nanoprobes. Assembly of aptamer-engineered SERS nanotags and MRS nanoprobes yields robust SERS performance and high transverse relaxation time (T2). Target aflatoxins induce separation of SERS nanotags and dispersion of Fe_3_O_4_@Au NPs, leading to decreases in SERS signals and T2 values. This platform offers a high-throughput strategy for simultaneous analysis of aflatoxin subtypes. Additionally, Tian et al. developed a novel biosensor combining SERS and FET modes for detecting MC-LR [101]. Using AuNPs/graphene composite, the SERS mode captured MC-LR Raman fingerprint via aptamer binding, with AuNPs enhancing the effect confirmed by simulations. The FET mode, employing a graphene FET (G-FET), achieved LODs of 0.62 aM in phosphate buffered saline (PBS) and 0.91 aM in human serum, with linear responses over a wide concentration range. The method may have promising applications in food matrix testing.

In summary, these studies demonstrate the broad applicability and significant potential of SERS when integrated with other detection technologies. Future research could adopt cross-modal alignment strategies from multimodal frameworks to enable joint modeling of SERS data with colorimetric, electrochemical, and fluorescence datasets, thereby enhancing multiplexed toxin detection.

### 4.6. SERS Multimodal Sensing

**Figure 8:**
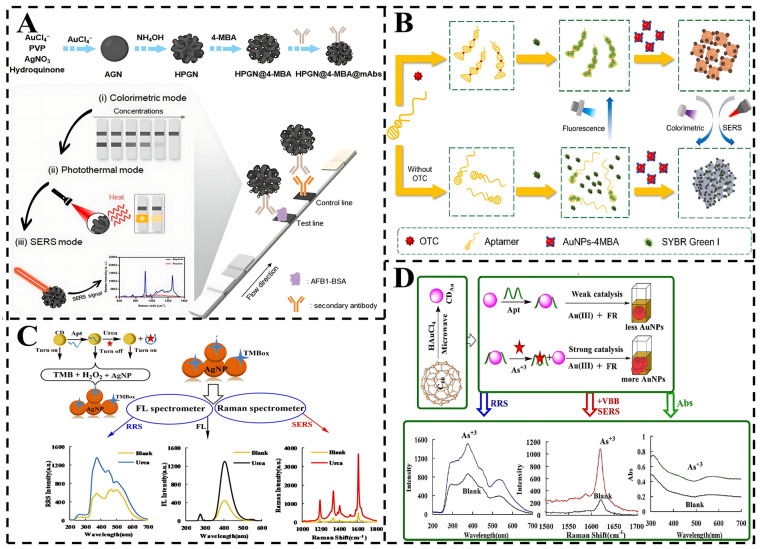
(**A**) Synthesis of HPGN@4-MBA@mAbs and its application in tri-mode LFIA of AFB1 [103]. (**B**) Schematic illustration of tri-mode detection of OTC based on the target-induced aptamer structure switching strategy [104]. (**C**) Schematic of SERS/RRS/FL tri-mode method to detect ultratrace urea coupling urea aptamer with CDFeN catalysis [105]. (**D**) Analytical principle of detection of As^+3^ by Apt-mediated nanocatalytic tri-mode molecular spectral method [106].

Multimodal sensing strategies incorporating three or more signal outputs exhibit strong anti-interference capabilities, meeting diverse testing requirements across different scenarios. The built-in cross-mode calibration significantly enhances result reliability. As a versatile analytical tool, SERS effectively complements fluorescence, colorimetry, and electrochemistry, enabling result visualization while expanding detection breadth and accuracy (Figure 8). Multimodal sensors can speed up the rate of preprocessing, connecting the signal unit to a smartphone for real-time dynamic monitoring using different detection modes in different scenarios [102]. Future implementations may achieve fully automated, low-sample-consumption, high-throughput screening of food toxins.

For example, Xie et al. synthesized a hollow porous gold nanoflower (HPGN). Compared with AuNPs, the HPGN showed excellent resistance to salt stress and a high photothermal effect [103]. Thus, a tri-mode method for colorimetric, photothermal, and SERS determination of AFB1 based on HPGN combined with LFIA (HPGN-LFIA) was constructed. The proposed HPGN-LFIA method can be used as a fast and simple multisignal platform for mycotoxin detection. Moreover, Shao et al. constructed a three-mode sensing system for the detection of OTC [104]. The system leverages OTC’s ability to induce conformational changes in aptamers, promoting the formation of duplex structures. SYBR Green I (SGI) is subsequently incorporated into these duplexes, enhancing the fluorescence signal. Additionally, OTC facilitates the aggregation of functionalized AuNPs-4MBA via charge neutralization, generating robust colorimetric and Raman signals. The LODs for the fluorescence, colorimetric, and Raman modes were found to be 0.074 nM, 5.019 nM, and 0.036 nM, respectively, with a broad detection range spanning five orders of magnitude (0.1–10,000 nM). When applied to tap water and honey samples, the method exhibited high analytical performance with recovery rates ranging from 90.11% to 119.75% (n = 3). This method demonstrates a wide detection range, ultralow LODs, and high specificity for toxin detection, making it a powerful tool for food safety monitoring.

Furthermore, Li et al. presented an Fe/N-codoped carbon dot (CDFeN) for the detection of urea. These dots effectively catalyze the oxidation of TMB by H_2_O_2_, generating an oxidized probe (TMBox) with distinct SERS, RRS, and fluorescence signals [105]. The urea aptamer can turn off these signals, enabling a novel aptamer-turn-on tri-mode method for ultratrace urea detection with high sensitivity and selectivity. By expanding the range of applications, Wang et al. successfully prepared Au-doped carbon dots (CDAu) using fullerenes as precursors. Notably, CDAu exhibited robust catalytic activity in converting HAuCl_4_ and fructose into AuNPs [106]. This novel nanocatalytic reaction was examined through SERS, RRS, and Abs spectrometry. Leveraging the specific aptamer-As^+3^ interaction to mediate the CDAu-HAuCl_4_-fructose nanoreaction and utilizing the resultant AuNPs as trifunctional indicators for SERS/RRS/Abs, a novel trimodal aptasensor strategy was devised for ultrasensitive As^+3^ detection. CDs, as a carbon-based nanomaterial, are recognized as green and biodegradable materials with potential applications for building multimodal sensors.

Table 2 comprehensively summarizes the performance of SERS-based multimodal biosensors for biotoxin detection. Multimodal biosensors utilize multiple complementary signal outputs for rapid on-site detection in a variety of scenarios and are characterized by high sensitivity, high specificity, high accuracy, wide adaptability, and cost-effectiveness. Through the integration of AI technology, sustainable materials, and super-resolution imaging, multimodal systems are poised to emerge as versatile platforms for comprehensive food safety monitoring.

## 5. Challenges and Future Perspectives

The advent of SERS multimodal sensors has revolutionized biosensing platforms by significantly improving accuracy through built-in cross-validation via multiple independent transduction mechanisms. Notably, SERS multimodal sensors exhibit enhanced sensitivity and contrast while reducing background interference compared to single-mode sensors, enabling precise quantification. With continued advancements, these sensors are expected to achieve laboratory-level performance while retaining the intrinsic advantages of SERS. However, there are still some problems with the design and practical application of SERS multimodal sensors. First, nanomaterials, with their universal properties, are key to the design of such sensors. Hybrid nanomaterials and smart nanomaterials that react with external stimuli allow for multiple signals to be produced, simplifying the design of sensors, but the sensitivity of the SERS depends heavily on the nanostructures used to enhance the Raman signal, and small changes in the nanoparticle synthesis, size, or shape may result in significant performance differences. The synthesis of stable, homogeneous, multifunctional nanomaterials is a challenge in ensuring reproducibility between different platforms and experiments. The successful fabrication of such ultrasensitive smart nanomaterials requires advanced techniques, including microfluidic chips and 3D printing technologies, to develop compact, multifunctional nanostructures.

Another critical challenge is that nanoprobes are prone to deactivation in complex food matrices, necessitating durability enhancement through inert encapsulation or self-healing materials to ensure stable signal output. For example, contaminants in food include proteins, enzymes, and metabolites. During long-term operation of implantable devices, biological contaminants will irreversibly adhere to their working surfaces and gradually alter the electrochemical and biological properties of these devices, leading to unpredictable device dysfunction and serious infections. This problem can be addressed through the development of sensor surface engineering and antifouling coating materials, such as biopolymers, hydrogels, porous Si, amphiphilic ion-modified proteins, etc. Additionally, enhancing sensitivity and specificity in food substrate is another hurdle, requiring advanced functionalization techniques and integration with machine learning to distinguish target signals from interfering molecules. Future research should aim at improving the selectivity of SERS probes by developing more sophisticated functionalization techniques, such as highly specific aptamers, antibodies, or peptide coatings for specific biomarkers. In addition, improved integration of SERS with machine learning algorithms helps to distinguish target signals from background noise for more accurate analysis of complex sample matrices.

In summary, integrating SERS with optical, electrical, and chemical sensing modalities into a unified analytical platform enhances data quality and generates robust results. This approach is exemplified by the successful merging of spectral and spatial data from hyperspectral imaging, which has demonstrated significant performance improvements. SERS multimodal sensors are poised to redefine diagnostic accuracy and monitoring efficacy, particularly in public health surveillance and food safety applications, enabling more precise and versatile analysis of complex food matrices. By addressing challenges related to signal stability, nanotechnology, interdisciplinary collaboration, machine learning, microfluidics, chip integration, and scalability, the future of SERS sensors holds not only great promise but also the potential for groundbreaking advancements, ultimately revolutionizing the way we detect and respond to threats in real-time and enhancing global health and safety standards.

## Figures and Tables

**Table 2:** Analytical performance of SERS-based multimodal biosensors for different biotoxins (Note: /, not reported).

Material	Target	Mode	Linear Range	LOD	Detection Time (T)/ Reproducibility (RSD or CV)/ Stability (S)	Real Samples	Recovery (%)	Reference
Colorimetric-SERS								
Au@HgNPs	OTA	Colorimetry	0.5–10.25 μg/L	0.29 µg/L	/	coffee	95.9–104.0	[107]
		SERS	0.25–18.75 μg/L	0.16 µg/L	/		96.7–108.9	
MIL-101/Au ETHH NRs	CAP	Colorimetry	32.3–3231.3 μg/L	0.48 μg/L	/	fish	95.64–110.62	[108]
		SERS	0.03–323.1 μg/L	3.23 ng/L	RSD: 6.7%		98.5–102.2	
Fe_3_O_4_@MOF-GNS@CP	CAP	Colorimetry	3.23–80,782.5 μg/L	6.69 μg/L	/	milk, honey, mineral	95.7–103.7	[109]
		SERS	0.32–323.13 μg/L	0.25 μg/L	RSD: 4.23%		91.4–103.6	
Fluorescence-SERS								
MXene-Au	Endotoxins	Fluorescence	0.1–2500 ng/mL	0.26 ng/mL	T: 30 min RSD: 1.6%	Water, milk	93.5–105.5	[110]
		SERS	0.01–2500 ng/mL	0.04 ng/mL	T: 30 min RSD: 4.8%		104.0–112.1	
AuNPs-4MBA/cDNA@MNPs-Cy5/AFB1	AFB1	Fluorescence	0.10–100 ng/mL	5.81 pg/mL	/	peanut	91.4–92.9	[111]
		SERS	0.10–100 ng/mL	0.01 ng/mL	/		91.7–95.6	
DTNB-AuNPs, QD@SiO_2_	AFB1	Fluorescence	0.0001–100 ng/mL	0.100 pg/mL	/	peanuts, peanut oil	95.22–105.80	[112]
		SERS	0.0001–1000 ng/mL	0.087 pg/mL	/		96.30–101.36	
AgNPs/MPDA-apt-TAMRA	DON	Fluorescence	0.1–100 ng/mL	0.08 ng/mL	RSD: 2.59%	wheat flour	98.00–104.1	[49]
		SERS	0.1–100 ng/mL	0.06 ng/mL	RSD: 1.37%		99.88–105.4	
AuNPs@PVP@RITC@SiO_2_NPs/ rGO-AuNS	T-2 toxin	Fluorescence	0.001–500 ng/mL	0.85 pg/mL	T: 25 min	corn, wheat	88.03–100.3	[113]
		SERS	0.001–500 ng/mL	0.12 pg/mL	T: 25 min		88.4–102.9	
MNP@Ag-PEI	AFB1	Fluorescence	0.2–20,000 ng/mL	0.135 ng/mL	/	peanut, walnut, almond	94.7–109.7	[114]
		SERS	0.001–1000 ng/mL	0.45 pg/mL	RSD: 3.60% S: 60 days		95.2–108.6	
cDNA-AuNPs, Apt-AuNSs	OTA	Fluorescence	1–100 ng/mL	0.17 ng/mL	/	coffee, wine	99.14–100.96	[115]
		SERS	5–250 pg/mL	1.03 pg/mL	/		99.85–118.08	
LFIA-SERS								
Au4-MBA@AgNPs	IMI, PYR, AFB1	SERS	0.025–1, 1–100, 0.025–0.25 ng/mL	8.6, 97.4, 8.9 pg/mL	T: 8 min RSD: 4.83%, 5.32%, 6.15% S: 28 days	Pu’er tea, black tea, surface water	91.40–112.50, 86.16–113.08, 90.40–115.00	[116]
PEC-SERS								
Au@Ag/H-WO_3_	MC-LR	SERS	1–100 ng/mL	0.13 ng/mL	RSD: 3.5% S: 7 days	lake water	90.0–96.0	[117]
		PEC	0.3–50 ng/mL	0.06 ng/mL	RSD: 2.3 % S: 7 days		97.0–98.0	
TLC-SERS								
ZIF-67/Ag NPs/Au NWs	MC-LR	SERS	4.976–497.6 ng/mL	2.259104 ng/mL	RSD: 10.4%	bellamya aeruginosa	93.28–101.66	[118]
Colourimetric/SERS/Fluorescent								
CRISPR/Cas12a, G4-DNAzyme	AFB1	Colourimetric	0.001–0.1 ng/mL	0.85 pg/mL		peanut, maize, badam	83.1–108.3	[119]
		SERS	0.001–0.1 ng/mL	0.79 pg/mL	RSD: 7.52% S: 7 days		85.9–106.8	
		Fluorescent	0.001–0.1 ng/mL	1.65 pg/mL			84.0–108.5	
SERS/Fluorescence/CD								
UCNPs	SEB	SERS	1–750 pg/mL	0.1 pg/mL	/	milk	80.93–105.45	[120]
		Fluorescent	1–750 pg/mL	0.1 pg/mL	/		85.27–106.26	
		CD	2–500 pg/mL	0.3 pg/mL	/		89.21–108.33	

CAP: Chloramphenicol; SEB: Staphylococcal enterotoxin B; IMI: Imidacloprid; PYR: Pyraclostrobin.

## Data Availability

No new data were created or analyzed in this study. Data sharing is not applicable to this article.
